# Dhurrin in Sorghum: Biosynthesis, Regulation, Biological Function and Challenges for Animal Production

**DOI:** 10.3390/plants13162291

**Published:** 2024-08-17

**Authors:** Bo Wang, Wangdan Xiong, Yanjun Guo

**Affiliations:** 1 Qingdao Key Laboratory of Specialty Plant Germplasm Innovation and Utilization in Saline Soils of Coastal Beach, Qingdao Agricultural University, Qingdao 266109, China; wangbo@qau.edu.cn (B.W.); xiongwd@qau.edu.cn (W.X.); 2 Key Laboratory of National Forestry and Grassland Administration on Grassland Resources and Ecology in the Yellow River Delta, Qingdao Agricultural University, Qingdao 266109, China; 3 College of Grassland Science, Qingdao Agricultural University, Qingdao 266109, China

**Keywords:** sorghum, dhurrin, hydrogen cyanide (HCN), animal production

## Abstract

Sorghum (*Sorghum bicolor*) holds a significant position as the fifth most vital cereal crop globally. Its drought resistance and robust biomass production, coupled with commendable nutritional value, make sorghum a promising choice for animal feed. Nevertheless, the utilization of sorghum in animal production faces hurdles of dhurrin (a cyanogenic glycoside) poisoning. While dhurrin serves as a protective secondary metabolite during sorghum growth, the resulting highly toxic hydrogen cyanide poses a significant threat to animal safety. This review extensively examines the biometabolic processes of dhurrin, the pivotal genes involved in the regulation of dhurrin biosynthesis, and the factors influencing dhurrin content in sorghum. It delves into the impact of dhurrin on animal production and explores measures to mitigate its content, aiming to provide insights for advancing research on dhurrin metabolism regulation in sorghum and its rational utilization in animal production.

## 1. Introduction

Sorghum, the fifth most prominent cereal, holds a pivotal position in global agriculture, following maize, rice, wheat, and barley. Endowed with drought tolerance and the capacity to thrive in regions restricted to other cereals, sorghum stands out as a prolific yielder, particularly in semi-arid and arid regions of the world [1]. Serving a multitude of purposes, sorghum caters to staple food needs (grain sorghum), livestock sustenance (grain, forage, and sweet sorghum), biofuel production (sweet, grain, and biomass sorghum), biogas generation (biomass sorghum), and syrup fabrication (sweet sorghum) [2]. Rich in carbohydrates, protein, vitamins, and minerals, sorghum grain presents a compelling alternative for human food and livestock fodder, boasting comparable protein content to wheat and exceeding maize and rice [3]. However, the presence of kafirin and a limited lysine content in protein hampers digestibility, while starch-rich grains exhibit reduced amylase activity and accessibility compared to wheat [4,5]. Anti-nutritional factors such as tannins and phytic acid impede nutrient assimilation, necessitating further exploration for efficient sorghum utilization. Furthermore, the shadow of cyanogenic glycosides, notably dhurrin, casts a pall over sorghum’s suitability as roughage [6,7,8]. Dhurrin, a key cyanogenic glycoside, acts as a defensive compound, deterring herbivores by converting to toxic hydrogen cyanide (HCN) upon hydrolysis, posing risks to animal health [9,10]. Unraveling dhurrin’s metabolic pathways presents opportunities to breed low-dhurrin sorghum varieties and maximize its role in animal production [11,12,13]. However, despite progress, many sorghum species retain cyanogenic glycosides, with synthesis heightened in young leaves and stems during vegetative growth [9]. The risk, influenced by species [14] and environmental factors [15,16], underscores the need for judicious selection, cultivation, and timing to mitigate HCN poisoning. In this review, we elucidate dhurrin’s metabolic pathways, pivotal genes in its regulation, and factors affecting dhurrin content. Moreover, we underscore sorghum grain and stalk efficacy for animal production, outlining potential strategies to curtail dhurrin content. This comprehensive overview aims to guide the spectrum of sorghum application, spanning cultivation to husbandry, with insights drawn from dhurrin metabolism and prudent utilization.

## 2. What Is Dhurrin

More than 3000 plant species are known to be cyanogenic in nature, such as the familiar crops cassava and sorghum. Dhurrin, one of the cyanogenic glucosides, is a secondary metabolite mainly produced by sorghum. For cyanogenic plants, dhurrin is one of the important self-protection systems to prevent biotic damage from animals, microorganisms, and humans by releasing a weak acid but seriously toxic substance, HCN. For invasive organisms, HCN mainly works on the mitochondria and interrupts mitochondrial oxygenic respiration [17]. The action from dhurrin to HCN is catalyzed by the hydrolytic enzymes (β-glucosidases), α-hydroxynitrile lyase and dhurrinase 1 or 2 [9,10,18]. Cyanogenic glucoside dhurrin (*β*-D-glucopyranosyloxy-(S)-*p*-hydroxymandelonitrile) is primarily produced in the leaves and in varying concentrations in leaf sheaths and culms of sorghum [19]. More specifically, it is mainly distributed in the epidermal, cortical, and vascular tissue, with the greatest amount in cortical tissue [20]. The extensive studies on sorghum have provided significant insights into the biosynthetic pathway and key genes involved in its production, making it the best-studied model for cyanogenic glucoside biosynthesis.

## 3. Role of Cyanogenic Glucosides (Dhurrin) for Plants

**Figure 1:**
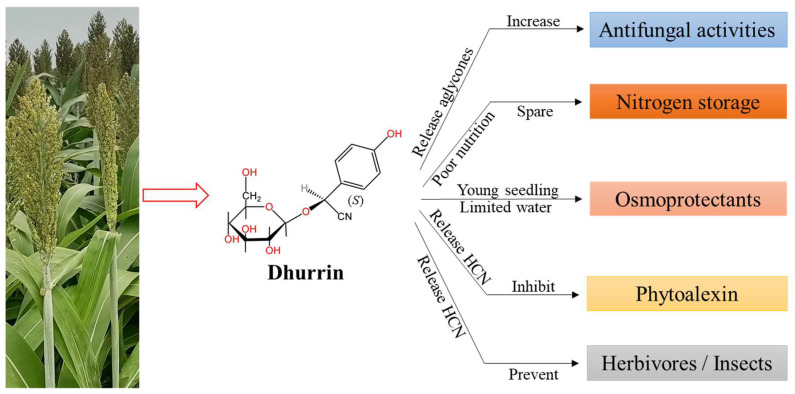
Function of dhurrin as part of the primary defensive mechanism in sorghum [23,27,28,29,30]. It serves several functions, including increasing some antifungal activities, storing nitrogen sources, acting as an osmoprotectant, inhibiting phytoalexin, and discouraging herbivores and insects.

Numerous plant genera possess the capacity to synthesize cyanogenic glucosides, which play a pivotal role as a primary defense mechanism in safeguarding plant growth and development (Figure 1) [21,22]. These compounds release aglycones that may contribute to the formation of chemical compounds with antifungal properties [23]. However, research has also indicated that certain fungi and insects show a preference for plants with higher levels of cyanogenic glucosides compared to those producing lower amounts [24]. This could be because they can hydrolyze cyanogenic glycosides by secreting specific enzymes, thus releasing hydrogen cyanide, which may help fungi survive and reproduce in competitive environments, although further confirmation is still needed. Furthermore, the liberation of HCN from cyanogenic glucosides might hinder the production of phytoalexins, potentially causing more harm to the plant than to the microorganisms [25,26]. Consequently, a comprehensive investigation is warranted to elucidate the response of plant cyanogenic glucoside synthesis to microorganisms and the intricate relationship between cyanogenic glucoside production, plant growth, development, and interactions with microorganisms and insects. Additionally, cyanogenic glucosides could serve other functions, such as acting as a nitrogen storage source in nutrient-poor environments among wild sorghum species [27,28] or functioning as an osmoprotectant when dhurrin is present in significant quantities within specific cell types or tissues, such as in young sorghum seedlings [29]. Notably, Burke et al. (2013) demonstrated substantial variation in leaf dhurrin content among sorghum lines, both before and after flowering, in a study assessing drought tolerance, suggesting that this metabolite could serve as an osmoprotectant when sorghum faces limited water availability [30]. Additionally, dhurrin may also influence plant–plant interactions by affecting competitive interactions among neighboring plants, but the opinion needs further study. For example, the presence of dhurrin in a plant’s tissues may confer a competitive advantage by reducing herbivore damage, allowing the plant to allocate more resources to growth and reproduction [21]. This can influence plant community composition and diversity, as dhurrin-containing plants may outcompete non-cyanogenic species in certain environments. In summary, cyanogenic glucosides emerge as pivotal metabolites crucial for preserving plant growth, particularly during instances of biotic or abiotic stress.

## 4. Biosynthesis of Dhurrin in Plants

### 4.1. Biochemical Synthesis and Catabolism Process of Dhurrin

**Figure 2:**
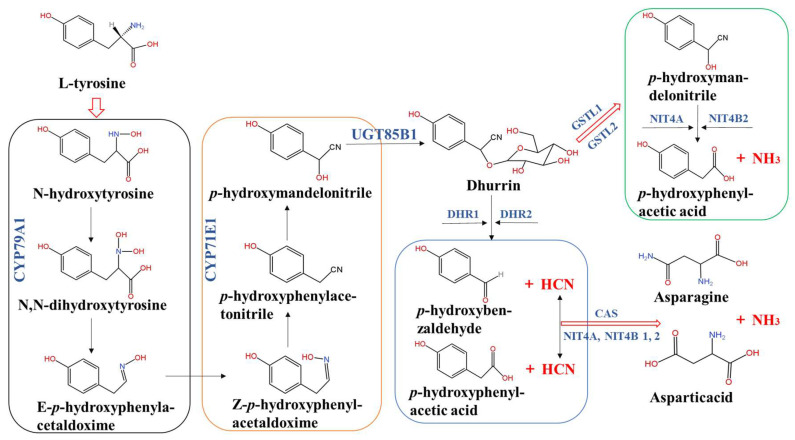
Biosynthesis and catabolism process of dhurrin in sorghum [13,29,31]. The key enzymes involved in the biosynthesis of dhurrin from L-tyrosine include CYP79A1, CYP71E1, and UGT85B1. The main enzymes participating in dhurrin catabolism include DHR1, DHR2, CAS, GSTL1/2, NIT4A, and NIT4B1/2.

Cyanogenic glucoside dhurrin (*β*-D-glucopyranosyloxy-(S)-*p*-hydroxymandelonitrile), primarily produced in the leaves and in varying concentrations in leaf sheaths and culms of sorghum [19], is the best-studied cyanogenic glucoside in sorghum. Dhurrin in sorghum is considered to take place at the cytosolic surface of the endoplasmic reticulum and stored in the acidic vacuolar compartment stably [12]. The pathway of dhurrin biosynthesis derived from L-tyrosine [29] and involves a number of unusual and labile intermediates, like an N-hydroxytyrosine, N,N-dihydroxytyrosine, E-*p*-hydroxyphenylacetaldoxime, Z-*p*-hydroxyphenylacetaldoxime, *p*-hydroxyphenylacetonitrile, and *p*-hydroxymandelonitrile (Figure 2). The biosynthesis pathway is catalyzed by two multifunctional membrane-bound cytochrome enzymes, P450 (CYP79A1 and CYP71E1) and a soluble UDPG-glucosyltransferase (UDP-Glc *p*-hydroxy mandelonitrile glycosyltransferase, UGT85B1) [21,31]. Firstly, tyrosine is converted to Z-*p*-hydroxyphenylacetaldoxime by the catalyzing of CYP79A1 through the intermediates as N-hydroxytyrosine, N,N-dihydroxytyrosine, and E-*p*-hydroxyphenylacetaldoxime. CYP79A1 demonstrates a remarkable degree of substrate specificity [11], and the initial step it catalyzes serves as the rate-limiting step in dhurrin biosynthesis [32]. Therefore, the content or activity of CYP79A1 holds significant importance in the regulation of dhurrin synthesis. Secondly, Z-*p*-hydroxyphenylacetaldehyde oxime is converted to *p*-hydroxymandelonitrile in the presence of CYP71E1. The substrate specificity of the CYP71E1 is lower than that of the CYP79A1 [11]. Lastly, *p*-hydroxymandelonitrile is converted to dhurrin under the action of UGT85B1. On the contrary, the catabolism of dhurrin includes two pathways: a toxic catabolic process and a non-toxic catabolic recycling pathway. Dhurrin can be degraded to release HCN (cyanogenesis process) as the toxic catabolism process and form the products *p*-hydroxybenzaldehyde and *p*-hydroxyphenylacetic acid by the catalysis of α-hydroxynitrile lyase and dhurrinase 1 or 2 (DHR1 or DHR2), respectively [9,10]. In addition, the produced HCN can be detoxified by β-cyanoalanine synthase (CAS) and nitrilases (NIT4A, NIT4B1, and NIT4B2) to retrieve the nitrogen of HCN for producing amino acids (asparagine and aspartic acid) and ammonia [13]. However, no HCN is produced in the non-toxic recycle pathway. Dhurrin is converted to *p*-hydroxyphenylacetonitrile by the enzymes of glutathione transferase (GSTL1 or GSTL2) and further metabolized to *p*-hydroxyphenylacetic acid and free ammonia by the enzymes of NIT4A and NIT4B2 using glutathione as a reducing agent [33].

### 4.2. Genes Involved in the Regulation of Dhurrin Metabolism

**Figure 3:**
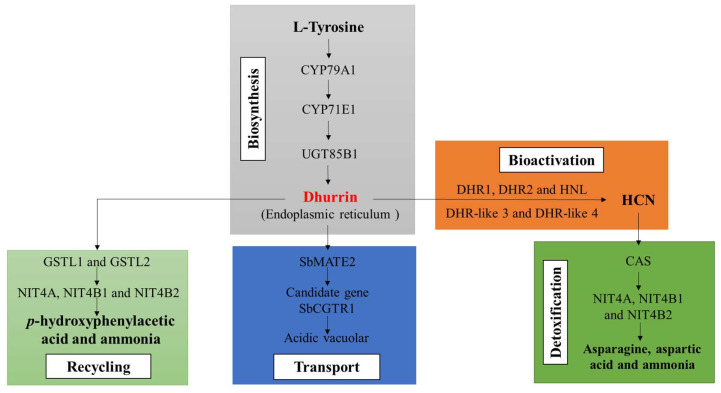
Key genes involved in the metabolism process [12,13,33,34,35,36,37]. The biosynthetic process of dhurrin from tyrosine is regulated by key genes such as *CYP79A1*, *CYP71E1*, and *UGT85B1*. The bioactivation process is primarily controlled by genes such as *DHR1/2*, *DHR-like3/4*, and *HNL*, which regulate the conversion of dhurrin to hydrogen cyanide (HCN). The detoxification process involves enzymes encoded by genes such as *CAS*, *NIT4A*, and *NIT4B1/2*, which convert HCN to asparagine, aspartic acid, and ammonia. The cycling process is facilitated by genes like *GSTL1/2*, *NIT4A*, and *NIT4B1/2*, which are responsible for transforming dhurrin into *p*-hydroxyphenylacetic acid and ammonia. The transport process is mainly carried out by *SbMATE2* and the candidate gene *SbCGTR1*, which is involved in the transport of dhurrin.

The genes governing the intricate process of dhurrin metabolism can be categorized into five distinct groups based on their roles in the biosynthesis, bioactivation, detoxification, recycling, and transport of dhurrin (Figure 3). The foundational understanding of the dhurrin biosynthesis pathway was established several years ago [29]. The pivotal players in dhurrin biosynthesis, namely *CYP79A1*, *CYP71E1*, and *UGT85B1*, are clustered on chromosome 1, orchestrating this process by encoding the relevant enzymes [10,12].

The bioactivation phase, involving dhurrin hydrolysis and the release of HCN alongside the co-product *p*-hydroxybenzaldehyde, involves five genes (*DHR1*, *DHR2*, *DHR-like3*, *DHR-like4*, and *HNL*), as gleaned from previous studies. Among these, the *DHR1*, *DHR2*, *DHR-like3*, and *DHR-like4* genes, positioned on chromosome 8, encode β-glucosidases (dhurrinases), while the *HNL* gene on chromosome 4 encodes α-hydroxynitrile lyase, collectively driving the catalysis of the bioactivation process [34]. Detoxification, a crucial aspect, relies on key genes like β-cyanoalanine synthase (*CAS*), along with the nitrilases *NIT4A*, *NIT4B1*, and *NIT4B2*. These genes operate to counteract auto-toxicity by detoxifying and reintegrating HCN into primary metabolic pathways like asparagine, aspartic acid, and ammonia [13]. The detoxification pathways of dhurrin facilitate the conversion of dhurrin into harmless metabolites, thereby preventing cyanide toxicity. Additionally, nitrilases *NIT4A*, *NIT4B1*, and *NIT4B2* partake in the dhurrin recycling process [35], while *GST* lambda candidates (*GSTL1* and *GSTL2*) crucially facilitate the conversion of dhurrin to *p*-hydroxyphenylacetic acid and ammonia without liberating HCN [33,36]. *SbMATE2*, a constituent of the multidrug and toxic compound extrusion (*MATE*) family, encodes a vacuolar transporter for dhurrin [12]. Furthermore, the nitrate/peptide family (*NPF*) transporter (*SbCGTR1*) in sorghum stands as a promising candidate for dhurrin transport [13]. It is noteworthy that *SbCGTR1* shares significant homology with a transporter identified in cassava, specifically designed for the transport of cyanogenic glucosides [37].

Although the key genes regulating dhurrin biosynthesis in sorghum have been identified for several decades, few transcription factors have been reported. Recent studies have found that *bHLH2* is involved in the amygdalin (one of the cyanogenic glucosides) biosynthetic pathway by controlling transcription of the P450 monooxygenase–encoding genes *PdCYP79D16* and *PdCYP71AN24* in almond [38]. Similar results demonstrated that *LjbHLH7* can directly activate the expression of the *CYP79D3* gene by binding to the G-box sequence of its promoter, and the *L. japonicus* Jasmonate-Zim Domain protein LjJAZ4 can act as a repressor of jasmonate-induced regulation of cyanogenic glucoside biosynthesis in *Lotus japonicus* [39]. A putative transcription factor, *SbGATA22*, may be involved in dhurrin biosynthesis by acting as a negative regulator of *SbCYP79A1* expression in sorghum [40]. Additionally, treating sorghum with MeJA (methyl jasmonate) also revealed some potential sequence motifs involved in the binding of transcription factors that result in increased expression of *SbCYP79A1* and the synthesis of dhurrin in sorghum [41]. As sequencing technology advances and sorghum genome annotation improves, it promises to unveil novel genes intricately involved in the regulation of dhurrin metabolism. This advancement will not only enhance our understanding but also present potential candidate genes for sorghum breeding endeavors.

## 5. Factors Affecting the Dhurrin Accumulation in Plants

Dhurrin, a compound with potential health risks, poses a significant constraint on sorghum’s utility for human consumption and animal feed. This concern has attracted the attention of researchers, consumers, and farmers alike. Dhurrin is predominantly found in the green parts of the sorghum plant, and its concentration is influenced by a range of factors. Notably, sorghum species, growth stage, and environmental conditions, specifically drought and nitrogen availability, emerge as pivotal determinants of heightened dhurrin levels.

### 5.1. Sorghum Species

Dhurrin content exhibits substantial variability among different sorghum species and varieties. Notably, the domesticated sorghum bicolor species exhibit considerably higher dhurrin content compared to the native wild species *Sorghum macrospermum* [42,43]. This distinction is reinforced by Cowan et al. (2022), who observed a higher dhurrin content in the leaves of *Sorghum bicolor* in comparison to wild sorghum species [44]. Interestingly, while differences in dhurrin levels were evident in leaf tissues, there were no significant variations observed in root tissues between the wild and domesticated *Sorghum bicolor* [45]. These differences may arise from distinct selective pressures experienced by domesticated and wild species within their respective natural and cultivated environments [46]. Additionally, the accumulation of dhurrin in the roots of wild species could potentially serve as a nitrogen reservoir, supporting growth in nutrient-depleted conditions typical of native habitats [47]. However, the underlying regulatory mechanism responsible for the marked difference in hydrogen cyanide content between wild and domesticated species exposed to similar environments remains unresolved. Moreover, an analysis involving 17 sorghum accessions revealed significant variation (ranging from approximately 6 to 22 µg/mg fresh leaf tissue) in dhurrin content, further underscoring the pivotal role of species in dhurrin modulation [48]. This diversity in dhurrin levels among sorghum species accentuates the challenges in selecting suitable species for large-scale cultivation and offers potential insights for breeding endeavors aimed at reducing dhurrin content for the production of safe sorghum for human and animal consumption. In the future, it will be necessary to build a database of dhurrin content in different varieties to provide a reference for breeding low-dhurrin sorghum varieties.

### 5.2. Growth Stage

Dhurrin concentration in sorghum is intricately linked to the plant’s growth stage and prevailing conditions [9]. It is present in all major tissues of domesticated sorghum species except for mature grains [49]. The biosynthesis and accumulation of dhurrin peak during the early growth stages of young sorghum seedlings and subsequently decline as the plant matures [50]. During germination and early seedling development, there is a rapid increase in the plant’s cyanide potential, followed by a decline due to the prevalence of dhurrin breakdown over its de novo synthesis as the plant matures. At its zenith, the cyanide potential can reach up to 60 nmol HCN per mg of plant material [9]. Additionally, during the young seedling phase, the content of cyanogenic glucosides may constitute a significant portion of the plant’s dry weight [29]. Conversely, the dhurrin content of the wild sorghum species *S. macrospermum* remains relatively stable throughout the seedling stage, with no significant fluctuations observed between day 10 and day 18 [43]. This divergence in dhurrin content dynamics between native and wild sorghum species could be attributed to variations in their functions and metabolic processes. In domesticated sorghum, cyanogenesis functions as a defense mechanism against herbivores [47]. However, the lower dhurrin content in wild species suggests an alternative reliance on defense mechanisms such as trichomes on leaf surfaces, with dhurrin potentially being converted into free nitrogen to support overall metabolic and growth processes rather than primarily serving for cyanogenesis [44]. Additionally, an investigation into dhurrin content across different developmental stages in 17 sorghum bicolor varieties unveiled four distinct patterns: (1) an increased dhurrin content during the vegetative stage compared to the seedling stage, followed by a decrease during the ripening stage; (2) a gradual decrease in dhurrin content across developmental stages; (3) a gradual increase from seeding to ripening stage; and (4) a significant decrease in dhurrin content during the vegetative stage compared to the seedling stage, followed by an increase during the ripening stage [48]. These diverse patterns underscore the complex interplay of genetic and environmental factors in regulating dhurrin content and further emphasize the differences between varieties, limiting the feasibility of utilizing young sorghum as forage due to variable dhurrin content.

### 5.3. Environmental Stress

In response to environmental stressors, plants initiate defensive measures to safeguard themselves. These responses encompass alterations in morphological, developmental, physiological, and biochemical traits [51]. Importantly, plants adjust their metabolic processes to produce functional metabolites, a vital strategy for survival under stress conditions. Amid abiotic stress factors, drought and nitrogen fertilization have been extensively investigated and elucidated.

#### 5.3.1. Drought Stress

Drought, a significant abiotic stressor, exerts pronounced effects on plant growth and development. Despite sorghum’s acknowledged drought tolerance and its capacity to counteract drought through mechanisms such as avoidance, recovery, survival, and tolerance [52], drought stress disrupts nutrient uptake from the soil and nutrient metabolism [53]. Dhurrin, a critical metabolite for assessing sorghum quality, is also impacted by drought stress. A pot experiment revealed that drought increased hydrogen cyanide accumulation and soluble protein content in the leaves of sweet sorghum cultivars, Sudangrass, and forage sorghum. Intriguingly, the exogenous application of abscisic acid and methyl jasmonate mitigated the drought’s effect on sorghum, resulting in enhanced plant weight and reduced HCN content. However, diverse physiological responses were noted across sorghum genotypes [54]. This elevation in dhurrin content under drought conditions has been supported by other studies [55,56]. Gleadow et al. (2016) further demonstrated a significant increase in cyanide concentrations in both leaves and stems of drought-stressed sorghum, with notably higher nitrogen allocation to cyanide in the stems of drought-stressed plants compared to well-irrigated ones [15]. Similarly, the response of dhurrin content to water stress (100% vs. 15% soil water capacity) differed between domesticated and wild sorghum species, underscoring the species-specific modulation of dhurrin content under drought conditions [43]. Additionally, pre-flowering and post-flowering drought tolerance in sorghum was correlated with dhurrin content before flowering, indicating the potential of dhurrin as a marker for drought tolerance [30]. The divergent dhurrin responses between cultivated and wild sorghum species to drought stress can be attributed to the same factors elucidated in the “Species” section, reaffirming the intricate interplay between species and environmental stressors in regulating dhurrin content. However, research on the mechanism of drought stress regulating dhurrin content in sorghum is still limited. Strengthening relevant studies can also provide insights into sorghum breeding strategies, which will help reduce dhurrin content and increase crop productivity [57].

#### 5.3.2. Nitrogen Fertilization

Nitrogen (N) represents a critical and limiting nutrient in crop production, particularly in low-quality soils. Effective nitrogen management significantly influences sorghum yield, N utilization, and quality [58,59]. Dhurrin, as part of the plant’s nitrogen pool, is also affected by nitrogen fertilization. The impact of available nitrogen on dhurrin content has been extensively explored. There are significant differences in dhurrin levels in sorghum under different nitrogen fertilizer levels, and the nitrogen levels allocated for dhurrin synthesis also vary. Sorghum grown under low nitrogen levels (1.5 mM) exhibited significantly lower HCN compared to those grown under medium (4.5 mM) and high (20 mM) nitrogen levels, resulting in average HCN concentrations of 0.13 mg/g, 0.43 mg/g, and 0.51 mg/g, respectively [56]. Moreover, a trade-off between nitrogen allocation to nitrate and dhurrin was observed, with excessive nitrogen favoring allocation to dhurrin before nitrate [15]. Interestingly, nitrogen allocation to dhurrin was more pronounced under limited nitrogen conditions, particularly at later developmental stages (from the eight-leaf to the ten-leaf stage) [60]. The activity of *CYP79A1* and *CYP71E1* genes, pivotal to dhurrin synthesis, was enhanced by nitrogen fertilizer application to sorghum plants aged 1 day to 5 weeks. This increase in gene activity coincided with elevated dhurrin content, although gene induction by nitrate occurred later in development [9]. Collectively, these findings underscore the intricate relationship between nitrogen availability, dhurrin content, and nitrate concentration. Further exploration of the allocation and utilization mechanisms of nitrogen fertilizer by sorghum at different growth stages, as well as the interactive effects of various environmental factors such as drought, waterlogging, and temperature on nitrogen fertilizer utilization, is promising for optimizing nitrogen utilization strategies for sorghum under various stress conditions.

## 6. Potential Impacts of Dhurrin in Sorghum on Animal Production

Sorghum, ranked as the fifth most significant cereal crop worldwide, finds application in various sectors, including human consumption, animal feed, industrial production, and alcohol manufacturing. Notably, two key types of sorghum, grain and forage sorghum, hold significant implications for animal production.

### 6.1. Sorghum Grain

Sorghum grain’s nutrient composition spans a wide spectrum: protein (6.2–14.9%), carbohydrates (54.5–85.2%), fat (1.3–10.5%), ash (0.9–4.2%), and fiber (1.4–26.1%) [61]. Globally, around 60 million tons of sorghum grain are produced annually, with a predominant share directed towards animal feed [5]. Approximately 35% of this production caters to human consumption, while the remainder serves animal feed, industrial applications, and alcohol production [62]. Dhurrin is either absent or present in minimal quantities in mature sorghum grain [63]. However, dhurrin accumulates during early grain development, peaking at about 25 days post-pollination, after which it is gradually replaced by proanthocyanidins during maturation [49]. Limited research exists on the impact of dhurrin in sorghum grain on animal feed.

However, several challenges hinder sorghum grain’s effectiveness as animal feed. Firstly, sorghum protein quality is suboptimal due to kafirins, which constitute about 70% of the grain’s protein content and are notably deficient in the essential amino acid lysine. Kafirins’ robust disulfide bridges hinder enzymatic digestion and protein assimilation, especially in conjunction with tannins and starch [5,64]. Secondly, although sorghum and wheat have comparable starch content, sorghum exhibits considerably lower amylase and amylolytic enzyme activity. Structural characteristics such as the peripheral endosperm layer surrounding starch granules, coupled with the inhibitory effects of phytate and tannin on α-amylase, contribute to sorghum’s diminished starch digestibility compared to other cereals [65]. Consequently, sorghum grain-based feed demonstrates reduced nutrient digestibility, potentially leading to risks of hydrogen cyanide poisoning if not fully matured.

### 6.2. Sorghum Forage

In the face of changing global climate patterns resulting in reduced rainfall, sorghum has emerged as a robust alternative to traditional forage crops like corn, particularly in arid regions. Its resilience to hot and dry conditions and higher biomass yield make it a valuable resource for animal production, particularly ruminants [66]. However, all parts of the sorghum plant contain dhurrin, a cyanogenic glucoside that releases toxic HCN when acted upon by specific endogenous β-glucosidases. Elevated dhurrin content in sorghum forage can lead to fatal cyanide poisoning in animals, with younger animals being more susceptible [67]. HCN disrupts the mitochondrial respiratory electron transport chain, inhibiting metalloenzymes in cytochrome *c* oxidase and causing severe detrimental effects on animal health [68].

Efforts to mitigate this risk have focused on breeding forage sorghum varieties with reduced HCN content and improved traits like dry matter yield, disease resistance, and nutrient quality [69]. Some studies demonstrate that sorghum silage, supplemented with corn meal, can replace corn silage without compromising milk yield and composition [70,71]. However, careful consideration must be given to dhurrin concentration in sorghum forage, as cyanide content decreases as sorghum matures. Ongoing efforts also aim to develop low-cyanide sorghum varieties [27,72]. Future research should explore the influence of dhurrin content on rumen fermentation, microbiota, nutrient absorption, metabolic processes, and quality of animal products to better understand sorghum forage’s potential in animal production.

## 7. Measures to Reduce Dhurrin Content in Sorghum

### 7.1. Breeding Strategies

Dhurrin, a natural defense against herbivores in forage plants like sorghum, is a target for reduction through selective breeding. The central aim of sorghum breeding is to comprehend dhurrin biosynthesis and minimize its presence in plants. The comprehensive sequencing and annotation of the sorghum genome, coupled with genetic studies like QTL mapping and BSAseq, have established a robust genetic foundation for enhancing sorghum bicolor for grain, forage, and bioenergy applications [73,74,75,76]. Despite the fact that no sorghum varieties with low dhurrin content have been reported yet, approaches such as molecular markers, introgression, and genome-wide association studies (GWAS) may be utilized to identify and select for low-dhurrin genotypes and contribute to more efficiently selecting plants with reduced cyanogenic glycoside content. A thorough examination of the dhurrin biosynthetic pathway in sorghum has identified CYP79A1 as a pivotal enzyme in catalyzing dhurrin synthesis [26,44]. Moreover, investigations have shown that dhurrin content in shoots of wild sorghum relatives is relatively lower compared to cultivated sorghum [44]. Myrans and Gleadow (2022) also found similar results and pointed out that in domesticated sorghum (in the subgenus Eusorghum), leaves can maintain high levels of cyanogenic glucosides (HCN potential > 0.4 mg g^−1^) until maturity, while this is not the case in wild taxa in other Sorghum subgenera [77]. Wild sorghum gene pools contain a large number of valuable genes for abiotic and biotic stress tolerance, and fully mining these genes has significant exploratory value for improving the resistance and yield of domesticated sorghum [78]. Therefore, the successful breeding of low-dhurrin sorghum varieties remains a challenge, but tapping into the genetic potential of wild sorghum relatives, manipulating dhurrin biosynthesis genes, and combining advanced breeding techniques hold promise for future sorghum breeding endeavors.

### 7.2. Production Management Approaches

Despite sorghum’s resilience to various stresses, limitations arising from the plant growth stage, environmental conditions, and soil fertility can lead to an undesirable increase in dhurrin concentration. The growth stage of sorghum emerges as a crucial factor influencing dhurrin content. As mentioned earlier, dhurrin levels in sorghum exhibit fluctuations across growth stages, typically declining gradually as the growth stage progresses. Notably, during sorghum grain maturation, dhurrin content experiences a rapid surge, peaking around 25 days after pollination and gradually reducing to minimal levels upon reaching maturity (67 days after pollination) [49]. Hence, when cultivating sorghum for animal feed, strategic timing for harvesting becomes vital—favoring pre-anthesis or post-grain maturity. This approach considers the varying concentrations of cyanogenic glycosides in different parts of the sorghum plant, including stems, leaves, and grains.

While sorghum is renowned for its drought tolerance, the pivotal role of water in determining yield and quality cannot be overlooked. Throughout its growth trajectory, sorghum’s development is affected by drought-induced stress, spanning from germination to reproductive and grain-filling stages. This stress triggers a cascade of effects on water use efficiency, transpiration rate, assimilate remobilization, and biochemical processes, ultimately leading to reduced grain yield and quality [79,80,81,82]. Importantly, dhurrin content, the primary hindrance to sorghum’s suitability as livestock forage, has been shown to increase under drought conditions, both in the early and late phases of sorghum development, rendering it unsuitable for animal consumption [15,27,56]. Therefore, whenever feasible, mitigating excessive water scarcity during sorghum’s growth cycle becomes crucial, creating conditions conducive to reduced dhurrin content and the cultivation of sorghum with lower dhurrin levels.

In the realm of sorghum production, the impact of fertilization extends beyond mere biomass accumulation, significantly affecting forage quality. Nitrogen, a fundamental element of fertilizers, has a dual effect on plant growth and nutritional value while also influencing dhurrin content. In the initial growth stage (up to 4 weeks), excessive nitrogen provision can lead to a preference for nitrogen allocation towards dhurrin synthesis [56]. Conversely, during the late growth stage (approximately eight- to ten-leaf stage), an increased allocation of nitrogen contributes to dhurrin synthesis when nitrogen resources align with sorghum’s normal growth requirements [60]. Consequently, the response of dhurrin content to nitrogen resources follows a distinct pattern across early and late growth phases. In a separate study, varying levels of fertilization (ranging from 75% to 125% of the recommended dose) revealed a direct correlation between increased fertilization and reduced HCN production, concurrently enhancing dry matter digestibility [83]. Employing a judicious fertilization strategy emerges as a path to promoting superior quality forage sorghum, characterized by reduced HCN content.

### 7.3. Processing Techniques

Among the primary techniques for preserving fodder, haymaking, and silage creation hold prominence. However, the efficacy of these methods in reducing HCN levels in sorghum forage varies. Notably, hay curing does not effectively reduce HCN, with a caveat: when HCN starts at relatively high but still sub-toxic levels (below 800 μg/g dry matter), the post-cutting metabolic activity could push HCN levels into the toxic range for animals [84]. Conversely, other studies present intriguing outcomes. In the context of cassava leaves, exposure to temperatures of 75 °C for 33 h led to the remarkable elimination of up to 99.95% of the original cyanide content [85]. Similarly, in a separate investigation, a sequence involving pounding, sun or shade exposure (2 h or 5 h, respectively), and triple water washing successfully reduced mean residual total cyanide content to a mere 1% [86]. While the effectiveness of hay curing against HCN poisoning risk in sorghum remains uncertain, methodologies derived from cassava leaf processing offer valuable insights for mitigating cyanide content in sorghum.

Fermentation stands as a potent means to significantly reduce cyanogenic glycoside content within plants. This transformative effect is exemplified by instances such as the reduction of 86.1% of cyanogenic glycosides (from 130.6 mg/kg DM to 18.1 mg/kg DM) through ensiling of *Acacia sieberiana* ground pods, achieving residual levels well below livestock safety thresholds [87]. Similarly, silage has proven effective in reducing HCN content by approximately 41.6% (from 506.8 mg/kg fresh weight to 295.8 mg/kg fresh weight) in cassava roots, though it still exceeds the safety threshold (200 mg/kg) for animal consumption [88]. Insights from ratooning sorghum indicate a reduction from 892 mg/kg to 407 mg/kg fresh weight of HCN content following 60 days of ensiling [89]. Despite these encouraging findings, the potential for residual poisoning risk in animal production persists when fermenting plants with initially high dhurrin content.

Significant progress has been made in identifying specific microorganisms capable of effectively reducing cyanogenic glycosides and HCN content. Pioneering investigations have highlighted food-grade fungi like *Neurospora sitophila* and *Rhizopus oryzae* as potent agents for cyanogen removal during solid cassava fermentation, attributed to their enhancement of cell wall degradation [90]. Additionally, *Bacillus subtilis* KM05 has exhibited an ability to engage in assimilatory degradation of cyanogenic glycosides in cassava flour [91]. Notably, *Zygosaccharomyces rouxii*, a prevalent yeast in *Prunus mume* syrup, has shown the capacity to reduce amygdalin concentration, similar to a cyanogenic glycoside, while potentially utilizing HCN as a carbon and nitrogen source or allowing its evaporation due to its volatile nature [92]. Most recently, the emergence of *Aspergillus niger* as a β-glucosidase producer has shown promise in the degradation of cyanogenic glycosides and subsequent HCN removal during sorghum fermentation, resulting in HCN levels below 100 mg/kg fresh weight and improved nutritive qualities [89]. Overall, while cyanogenic glycosides posed a challenge to sorghum’s utility, leveraging fermentation and targeted microorganism introduction offers a pathway to mitigate their levels, thereby enhancing the safety of sorghum for animal production. Continued research and innovation in this field are essential for addressing the challenges associated with dhurrin toxicity and ensuring the sustainability of sorghum production for animal consumption.

## 8. Conclusions

**Figure 4:**
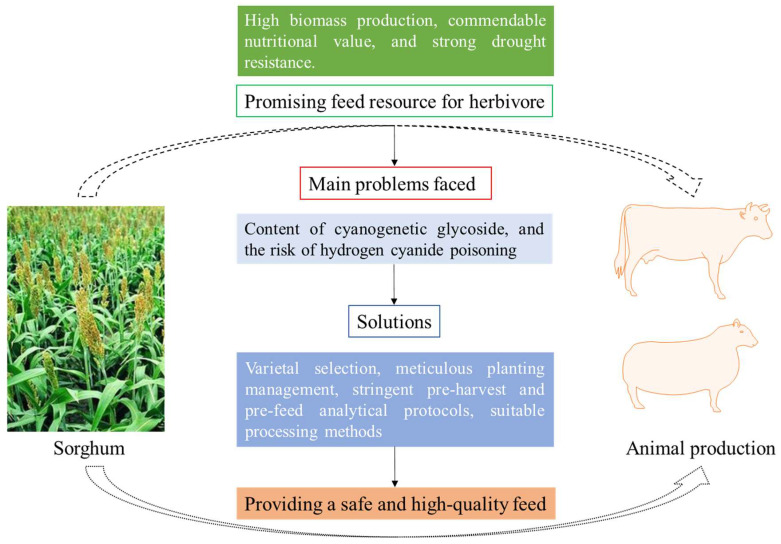
Measures to improve sorghum as a feed for herbivores. By adopting methods such as breeding, planting management, stringent analytical protocols, and suitable processing technologies, the risk of hydrogen cyanide poisoning when using sorghum as a forage resource for ruminant animals can be effectively addressed.

Sorghum stands as a highly promising feed resource for animal production, especially under limited environmental conditions that pose challenges for other staple crops like maize and wheat. Nevertheless, the presence of dhurrin remains a critical hurdle impeding the extensive utilization of sorghum as animal feed, primarily due to the inherent risk of HCN poisoning. In navigating this landscape, careful considerations must be made during sorghum cultivation, encompassing variety selection, meticulous planting management, and stringent pre-harvest and pre-feed analytical protocols targeting dhurrin and HCN levels, as well as suitable processing methods (Figure 4). By undertaking these measures, the menace of HCN poisoning can be effectively mitigated.

To foster continual advancements in sorghum’s suitability as livestock fodder, focused efforts are imperative. A deepened understanding of the regulatory pathways governing dhurrin metabolism is paramount. Likewise, unraveling the functions of key genes engaged in these regulatory pathways is crucial for informed interventions. Enhanced management strategies across the spectrum of sorghum production processes are vital as well. By synergizing these multi-faceted endeavors, the ambition is to cultivate sorghum that seamlessly aligns with the stringent requirements of livestock, ultimately realizing its potential as a safe and high-quality feed option.
